# Early immune response to *Coccidioides* is characterized by robust neutrophil and fibrotic macrophage recruitment and differentiation

**DOI:** 10.1128/spectrum.00442-25

**Published:** 2025-07-24

**Authors:** Nadia Miranda, Oscar A. Davalos, Aimy Sebastian, Margarita V. Rangel, Nicole F. Leon, Bria M. Gorman, Deepa K. Murugesh, Nicholas R. Hum, Gabriela G. Loots, Katrina K. Hoyer, Dina R. Weilhammer

**Affiliations:** 1 Quantitative Systems Biology Graduate Program, University of California Merced 33244, Merced, California, USA; 2 Biosciences and Biotechnology Division, Lawrence Livermore National Laboratory 4578, Livermore, California, USA; 3 Department of Orthopedic Surgery, University of California Davis Health 70083 , Sacramento, California, USA; 4 Department of Molecular and Cell Biology, School of Natural Sciences, Health Sciences Research Institute, University of California Merced 118577, Merced, California, USA; Geisel School of Medicine at Dartmouth, Lebanon, New Hampshire, USA

**Keywords:** *Coccidioides*, scRNAseq, neutrophils, macrophages, Valley fever

## Abstract

Coccidioidomycosis, or Valley fever, is an emerging respiratory disease caused by soil-dwelling fungi of the *Coccidioides* genus that is expected to spread from the southwest into the central U.S. by 2050. While 60% of infections are asymptomatic, the other 40% of patients experience a range of symptoms, from self-limiting pneumonia to life-threatening disseminated disease. The immunological events that underlie the progression to severe disease remain underdefined. Here, we probed the early immune response to *Coccidioides* using a high dose of an attenuated strain of *Coccidioides posadasii* in a mouse model of infection coupled with single-cell RNA sequencing. At 24 h post-infection, robust immune infiltration is detected in the lung, marked by high levels of inflammatory PD-L1^+^ neutrophils and fungal-contact-dependent pro-fibrotic Spp1^+^ macrophages. These findings elucidate the early dynamics of the host response to *Coccidioides* and provide a deeper understanding of host-pathogen interactions in the lung.

**IMPORTANCE:**

By examining early immune dynamics in the lungs, we uncover critical insights into how myeloid cells, particularly neutrophils and macrophages, are recruited and differentiated during *Coccidioides* infection. The discovery of specific immune cell subsets, such as PD-L1^+^ neutrophils and Spp1^+^ macrophages, which are associated with inflammation and fibrosis, highlights potential targets for therapeutic intervention. These findings provide a deeper understanding of the host-pathogen interactions that occur during *Coccidioides* infection, offering valuable directions for developing more effective treatments and preventive strategies against this increasingly prevalent disease.

## INTRODUCTION

Climate change is accelerating the spread of fungal infections due to rising temperatures and shifting environmental conditions (1). These changes are expanding the habitats of fungi, transforming previously non-endemic areas into new hotspots for infections, thereby increasing the risk to humans, pets, and livestock (2). This expanding geographic range of fungi highlights the urgent need for preventative measures to combat the rising threat of fungal diseases. One such emerging fungal infection is *Coccidioides*, an airborne pathogen that is progressively spreading from the southwest toward the central United States due to climate change (3).

Coccidioidomycosis, or Valley fever, is caused by inhaling the fungi *Coccidioides immitis* or *Coccidioides posadasii* in airborne dust (3). Infection outcomes vary widely, with the majority (~60%) of exposed individuals resolving the infection asymptomatically, whereas the remaining ~40% of individuals will develop disease that resembles bronchitis or pneumonia of varied severity (4). A subset of patients (~5%) will develop severe, disseminated disease that requires lifelong antifungal treatment, which can be fatal if untreated (4). *Coccidioides* infections continue to rise each year in California and Arizona, states with mandatory reporting, while the disease is underestimated in other southwestern states where reporting is not mandatory. Efforts to reduce the impact of Valley fever have been limited due to the lack of a definitive and rapid diagnostic, an insufficient understanding of the factors that predict which patients will develop severe disease, and a lack of effective therapies for those who experience disseminated disease. Thus, there is an urgent need to define effective host immunity to develop protective vaccines and immune-modifying therapeutics, as well as identify diagnostic targets.

*Coccidioides* spp. are dimorphic fungi that alternate between saprobic and parasitic life cycles in response to environmental conditions. Infectious arthroconidia are produced during the saprobic cycle in soil and can become airborne when the soil is disturbed. Once inhaled, they rapidly initiate the parasitic life cycle and start the transition to immature spherules within 24 h (5). As the fungus differentiates, host cells within the lung are exposed to different molecules of varying immunogenicity (6, 7). Arthroconidia are initially wrapped in a hydrophobic rodlet layer that masks underlying immunostimulatory ligands, limiting immune recognition until the rodlet coat sloughs. As the arthroconidia swell and begin the transformation to early spherules, host cells are exposed to β-glucan, spherule outer wall glycoprotein, and chitins, which are potent Dectin-1, Dectin-2, and toll-like receptor 2 ligands that drive early innate immune recognition (6, 8–10).

The mechanistic details that underlie the early interactions in the lung with *Coccidioides*, including the transcriptional changes induced within host cells and how those changes shape downstream antifungal responses, remain underdescribed. Among the earliest responders to infection are neutrophils and other myeloid populations such as monocytes and macrophages (6). Neutrophils have a complex role in *Coccidioides* infection (11–16). They are crucial for vaccine-induced immunity but do not alleviate fungal burden in unvaccinated mice (17, 18). Monocytes recruited from circulation rapidly differentiate into macrophages upon migrating into infected regions of the lung, where they produce tumor necrosis factor-alpha (TNF-α), interleukin-6 (IL-6), and reactive oxygen species in response to *Coccidioides*, key hallmarks of an innate inflammatory response (19, 20). The contribution of barrier epithelial cells as well as other resident cell types of the lung to the initial antifungal response, and how the coordinated effort between resident and infiltrating cells drives the differentiation of effector populations with unique functions specific to the invading pathogen (21), remains unknown in the context of *Coccidioides* infection.

Here, we utilized single-cell RNA sequencing (scRNAseq) and fluorescently labeled fungal spores to probe the early immune response within the lung elicited against a high dose of the live attenuated *C. posadasii* (Δcts2/Δard1/Δcts3) strain. This strain’s attenuation results from the deletion of chitinase genes (CTS2 and CTS3) and the ARD1 gene, producing sterile spherules that fail to progress through the parasitic life cycle, although early steps in the transition of arthroconidia to spherules remain intact and proceed similarly as wild-type strains (22). Early events in the lung following a physiological dose with wild-type fungus are difficult to trace due to the low concentration used (50–500 spores typically) and the resulting rarity of direct host-pathogen interaction events. Thus, a high dose of the attenuated strain is a useful tool for studying the earliest interactions between host cells and fungus *in vivo*. Within 24 h post-infection (hpi), there is a large influx of neutrophils and macrophages into the lungs. Diverse populations of neutrophils emerge, including a population that expresses high levels of *Cd274*, the gene that encodes PD-L1. Monocyte-derived Spp1^+^ macrophages (Spp1^+^ Mac) differentiate in a fungal contact-dependent manner and express a pro-fibrotic gene signature. Labeled *Coccidioides* preferentially associates with neutrophils and Spp1^+^ macrophages, as revealed by tissue imaging and flow cytometry. This multi-omics approach, incorporating flow cytometry, sequencing, and fungal imaging, provides novel insights into the early immune response against *Coccidioides*.

## RESULTS

### Single-cell RNA sequencing reveals robust myeloid cell infiltration into the lungs concurrent with shifts in non-immune cells

**Fig 1:**
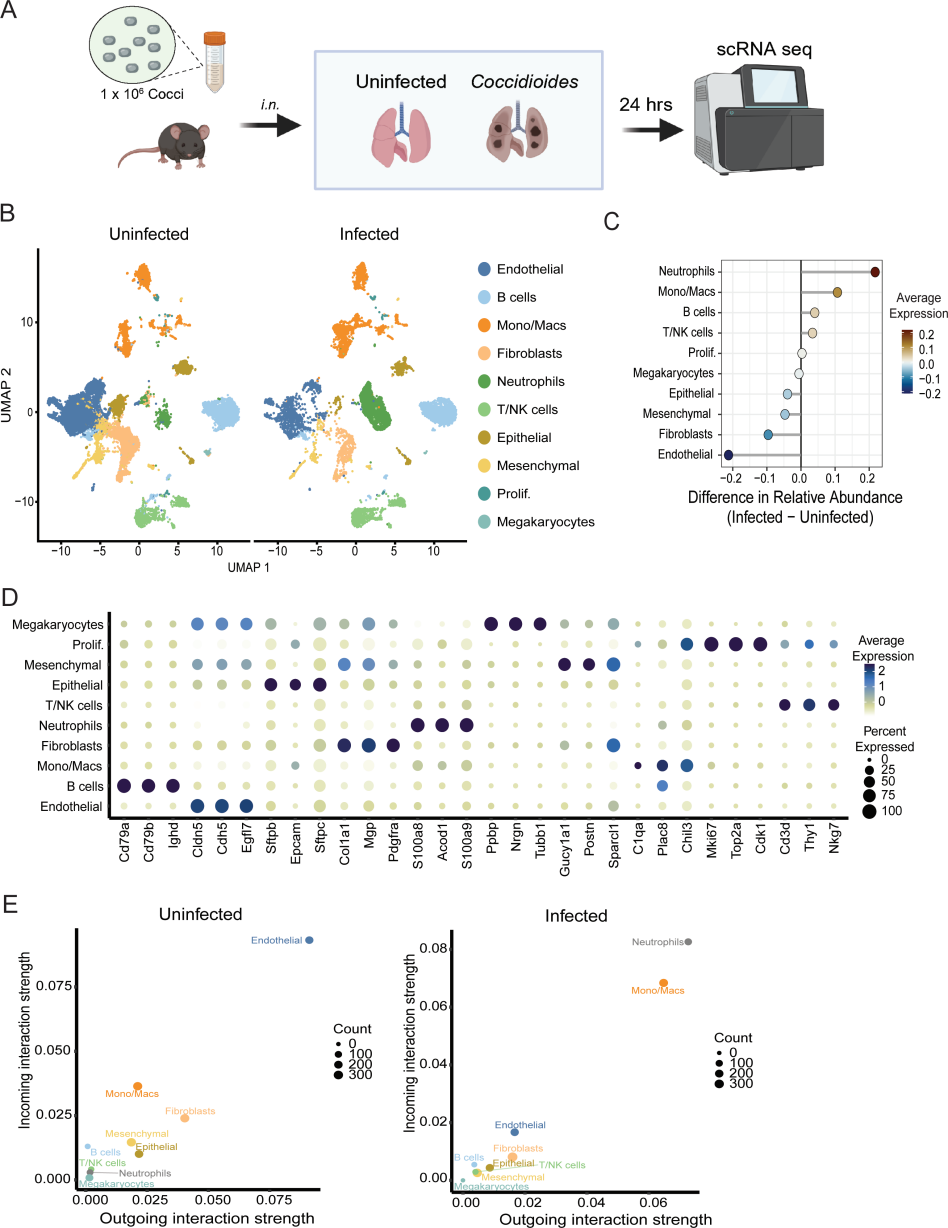
scRNAseq reveals shifts in cell populations and immune infiltration in the lungs following *C. posadasii* infection. (**A**) Experimental design. Mice were infected intranasally with 1 × 10^6^ *C. posadasii* (Δcts2/Δard1/Δcts3) arthroconidia or mock infected. Lungs (*n* = 3 pooled per condition) were harvested for scRNAseq analysis 24 hpi. (**B**) Uniform manifold approximation and projection (UMAP) visualization of cell clusters identified in each experimental group. (**C**) Relative cellular proportions in infected vs uninfected lungs. (**D**) Dot plot showing the expression of select cell-type markers. Dot size represents the fraction of cells of the indicated cluster expressing the markers, and the intensity of color represents the average marker expression level in that cluster. (**E**) CellChat analysis of incoming and outgoing signal strength in the uninfected and infected groups from cell populations in panel **B**. Dot size indicates the number of interactions. *n* = 3 pooled per condition for sequencing.

To elucidate early events in *Coccidioides* infection*,* we used scRNAseq to profile the transcriptional response in the lung in both immune and non-immune cells. C57BL/6 mice were intranasally infected with *C. posadasii* Δcts2/Δard1/Δcts3, and lungs were harvested 24 hpi, along with mock-infected controls (Fig. 1A). Unsupervised clustering of 16,265 and 9,990 cells from uninfected and infected lungs resulted in 12 clusters that were each assigned to a putative cell-type identity based on their unique profile of differentially expressed genes (DEGs) encoding cell-type-specific markers (Fig. 1B and D; Fig. S1A). The relative proportion of several immune cell populations increased upon infection, most notably neutrophils and monocytes/macrophages, along with a reduction in the relative proportion of non-immune cells such as endothelial cells, epithelial cells, and fibroblasts (Fig. 1C). To further understand cellular interactions, CellChat was utilized to examine cellular communication and signaling pathways involved during infection (23). Within uninfected lungs, endothelial cells were the dominant cell type in terms of incoming and outgoing signaling strength (Fig. 1E). Consistent with their increased abundance in the lung, neutrophils and monocyte/macrophages dominate cellular communication in the infected lungs, with a notable shift in signaling strength in both the incoming and outgoing interactions as well as an increase in the number of interactions (Fig. 1E), suggesting significant reorganization of cellular communication networks in the lung in response to the robust neutrophil and macrophage infiltration.

**Fig 2:**
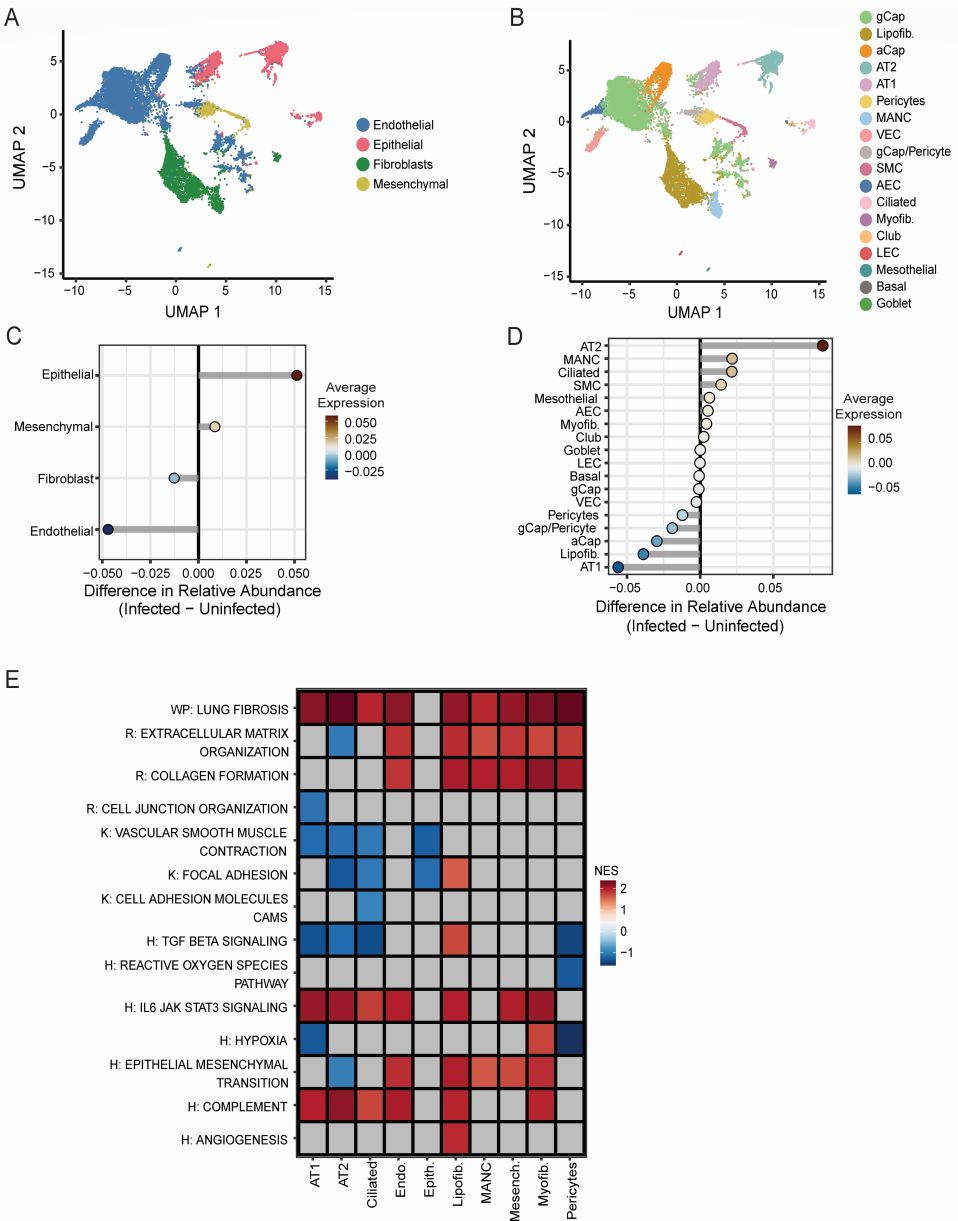
Non-immune cells respond to infection by upregulation of select inflammatory and pro-fibrotic pathways. (**A**) Integrated uniform manifold approximation and projection (UMAP) of the main non-immune cell types of the lung. Cells are color coded by type. (**B**) Integrated UMAP of subclustered non-immune cell types of the lung. Cells are color coded by type. Relative cellular proportions of main non-immune cell types (**C**) and subclustered non-immune cell types (**D**) in infected vs uninfected lungs. (**E**) Heatmap of pathway activities scored per cell by AUCell. Enrichment is represented by a scaled average AUCell score per cell type, with blue indicating lower enrichment and red indicating higher enrichment. The *X*-axis denotes the cell type, color coded by non-immune cell subtype as in panel **A**, and the *Y*-axis denotes the pathway.

Endothelial, epithelial, fibroblast, and mesenchymal lung cells maintain homeostasis within the lung microenvironment and are among the first cell types to encounter invading pathogens. To probe the response of these resident lung cells to infection, we subclustered the non-immune cells, resulting in 18 distinct cell clusters (Fig. 2A and B; Fig. S1B and C). Although the relative abundance of all non-immune cell types decreased with infection (Fig. 1C), epithelial cells increased in abundance relative to other non-immune cell types, while endothelial cells decreased (Fig. 2C). The increase in epithelial cells was primarily due to an expansion of alveolar type 2 (AT2) and ciliated cells, while club and goblet cells remained the same and alveolar type 1 cells decreased. Mesenchymal alveolar niche cell fibroblasts increased slightly in abundance, while myofib remained steady, and lipofib decreased. Endothelial subtypes either remained steady or decreased. The proportion of all mesenchymal cell subtypes remained steady. These data are summarized in Fig. 2D.

To gain deeper insights into the pathways active within the non-immune subpopulations, we performed an enrichment analysis by identifying differentially expressed genes in infected cells vs uninfected cells. Strikingly, several pathways involved in fibrosis, including lung fibrosis, collagen formation, epithelial mesenchymal transition, and extracellular matrix organization pathways, were enriched widely across many cell types, including fibroblasts, endothelial cells, mesenchymal, and some subsets of epithelial cells (Fig. 2E). IL-6/JAK/STAT3 signaling and complement were also enriched across multiple cell types. These findings highlight a shift toward profibrotic and inflammatory pathways within non-immune cells in response to infection.

### Diverse neutrophil populations respond to infection

**Fig 3:**
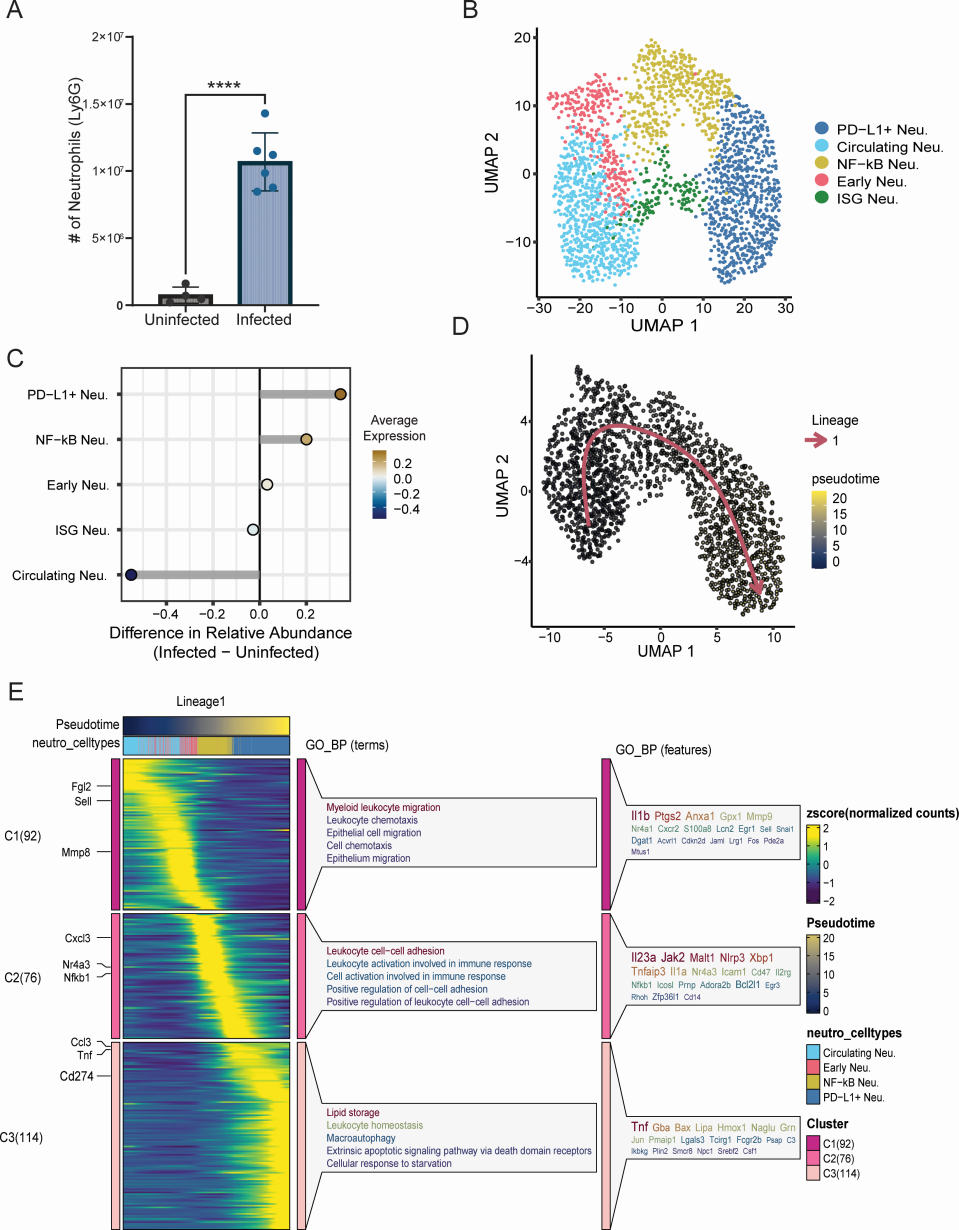
Diverse neutrophil populations accumulate in the lung 24 hpi. (**A**) Number of neutrophils in the lungs as identified by flow cytometric analysis. (**B**) Integrated uniform manifold approximation and projection (UMAP) of subclustered neutrophils. Cells are color coded by type. (**C**) Relative cellular proportions of neutrophil subsets. (**D**) UMAP plot displaying pseudotime trajectory. Cells are color coded based on their pseudotime values, ranging from blue (early) to yellow (late). The pseudotime trajectory was inferred in an unsupervised manner using Slingshot. (**E**) Heatmap illustrating the scaled fitted gene expression values along pseudotime. Expression levels are color coded from low (purple) to high (yellow). The top row for lineage represents pseudotime, ordered from left to right and color coded based on pseudotime values, ranging from blue (early) to red (late). The next row contains cell type colors, where each bar represents a cell belonging to a specific cell type along the pseudotime trajectory. Rows represent genes, with selected genes highlighted on the left-hand side. Genes were clustered based on fitted expression values, with each cluster represented by row-wise breaks in the heatmap. Each cluster is labeled on the left-hand side by “C#” followed by the number of genes in that cluster in parentheses. Different gene clusters can be distinguished by the clusters on the left-hand side of the heatmap, represented by a color block. Genes from each cluster were then analyzed for GO enrichment, with the top five enriched terms for each cluster shown on the right side of the heatmap. ****P < 0.0001.

Neutrophils were highly enriched in infected samples (Fig. 1C), and a significant increase in neutrophils within the lungs of infected mice was additionally confirmed by flow cytometry (Fig. 3A). To probe the diversity of the neutrophil (neu) response, 2021 cells were extracted and re-clustered, revealing five distinct subtypes: Circulating neu (Cxcr4, Sell, and Fgl2), Programed death-ligand 1 (PD-L1) neu (*Cd274*, *Ccl3*, and *Tnf*), NF-кB high neu (*Nfkb1*, *Cxcl3*, and *Nr4a3*), Early neu (*Mmp8*, *Retnlg*, and *Ngp*), and interferon-stimulated gene (ISG) expressing neu (*Rsad2*, *Ifit3*, and *Ift3b;* Fig. 3B; Fig. S2A through C). Circulating neu were the predominant subtype in the lungs of uninfected mice, and a dramatic shift toward PD-L1 and NF-кB neu was observed following infection (Fig. 3C; Fig. S2B). Gene ontology (GO) analysis demonstrated that neutrophil subpopulations were enriched for distinct pathways (Fig. 3E; Fig. S2D). Circulating, early, and NF-кB neu were enriched for pathways associated with chemotaxis, migration, and cell-cell adhesion, with early neu further enriched for pathways associated with response to reactive oxygen species/oxidative stress and NF-кB neu demonstrating enrichment for cellular activation/signaling pathways (Fig. 3E; Fig. S1D). ISG neu were distinctly enriched for pathways associated with response to viruses, and PD-L1 neu demonstrated overlapping enrichment characteristics with NF-кB and ISG neu, enriched primarily in pathways related to cell activation and signaling (Fig. 3E; Fig. S1D). Consistent with a shift toward a more activated phenotype upon infection, an increase in TNF and complement signaling between neutrophils and several immune and non-immune cell types was observed, most notably with monocyte/macrophages (Fig. S2E and S3A).

To better understand the differentiation of infection-induced neutrophil subsets along with genes changed over the lineage, we leveraged Slingshot (24) to perform a pseudotime trajectory analysis. Initial analysis identified two neutrophil lineages (data not shown). However, as ISG neutrophils were largely unchanged during infection (Fig. 3C), this population was excluded from subsequent analysis. One neutrophil differentiation lineage was identified, indicating that neutrophils differentiated in a trajectory through circulating, early, NF-кB-expressing, and ultimately into PD-L1^+^ neutrophils (Fig. 3D). Gene expression patterns clustered into three groups, and GO analysis of these clusters demonstrated different biological activities associated with each group (Fig. 3E; Fig. S2D). Clusters 1 and 2 (C1 and C2) contained circulating and early neu, showing enrichment for migration and IL-1β responses. C2 also demonstrated enrichment for cell-cell adhesion, activation, and IL-23, and was primarily composed of NF-кB neu. C3 displayed high expression of TNF, PD-L1, lipid storage, and macroautophagy, marking the termination of the neutrophil lineage at PD-L1^+^ neutrophils (Fig. 3D and E). These data indicate a dynamic neutrophil response occurring early following high-dose *Coccidioides* infection.

### Monocyte-derived Spp1^+^ macrophages exhibit a fibrotic state

**Fig 4:**
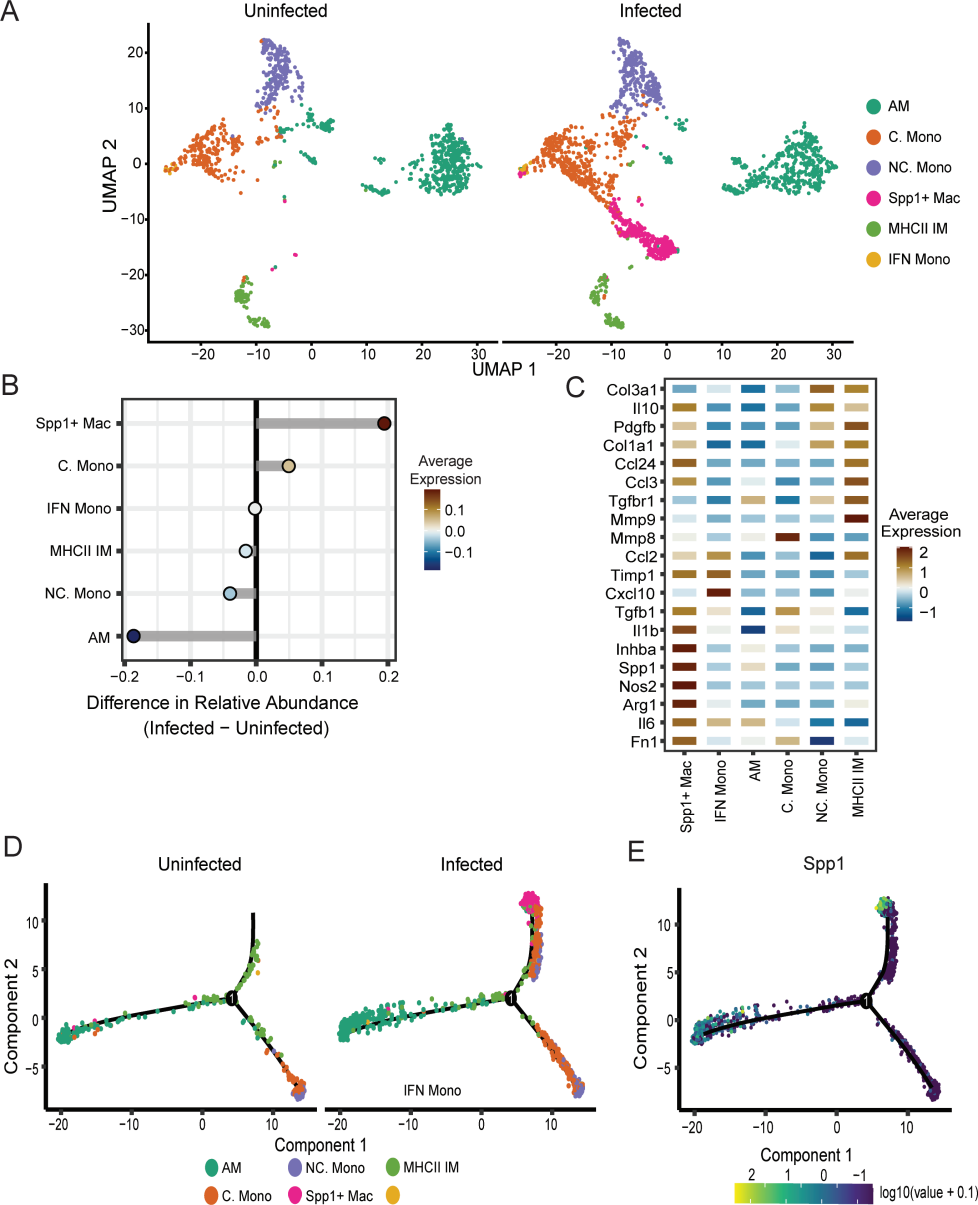
Spp1^+^ macrophages emerge following infection and display a fibrotic phenotype. (**A**) Uniform manifold approximation and projection (UMAP) visualization of cell clusters identified in each experimental group. (**B**) Relative cellular proportions of monocyte/macrophage subsets. (**C**) Heat map showing expression level of select fibrotic and inflammatory signaling genes across monocyte and macrophage subtypes. Trajectory analysis on monocyte/macrophage subclusters, colored by identity (**D**) or *Spp1* expression (**E**).

Monocyte/macrophage (Mono/Mac) cells were also highly enriched in infected samples. To gain deeper insights into their expression profiles, the Mono/Mac cluster (Fig. 1B), totaling 2,599 cells, was extracted and re-clustered, resulting in five subclusters: alveolar macrophages (AM; *Plet1*, *Krt19*, and *Lpl*), classical monocytes (*Plac8*, *Ly6c2*, and *Sell*), non-classical monocytes (*Adgre4*, *Ace*, and *Treml4*), Spp1^+^ Mac (*Fn1*, *Spp1*, and *Inhba*), MHC II high interstitial macrophages (MHCII IM; *Cd163*, *H2-Ab1*, and *C1qa*), and interferon high monocytes (IFN Mono; *Rsad2*, *Ifit2*, and *Ifit3*; Fig. 4A; Fig. S3D). The Spp1^+^ macrophages represent a novel subclass that emerges only upon infection and display the highest relative abundance within infected lungs (Fig. 4B). In response to *Coccidioides* infection, Spp1^+^ macrophages express high levels of pro-inflammatory (*Nos2*, *Tgfb1*, *Il1β*, and *Il6*) and fibrotic/wound healing associated genes (*Inhba*, *Spp1*, *Arg1*, *Pdgfb*, and Fn1; Fig. 4C). MHC II IMs express angiogenesis (*Col3a1* and *Pdgfb*), pro-inflammatory (*Ccl3* and *Mmp9*), and fibrotic markers (Ccl24). CellChat analysis revealed that *Spp1* signaling from Mono/Macs toward neutrophils was elevated in infected lungs (Fig. S3E).

To understand macrophage polarization, trajectory analysis was applied to the Mono/Mac clusters on the combined uninfected and infected data, revealing three distinct lineages (Fig. 4D; Fig. S3C). Lineage one consisted of classical and non-classical monocytes. Lineage two was composed of alveolar macrophages, reflecting a distinct lineage from other subsets. Lineage three encompassed MHC II IM in uninfected samples and expanded to include monocyte populations and Spp1^+^ Macs in infected samples (Fig. 4D). Pseudotime analysis indicated that classical and non-classical monocytes move toward the Spp1^+^ lineage after infection (Fig. S3C), and *Spp1* expression is highest at the termination of lineage three (Fig. 4E). Interestingly, moderate expression of *Spp1* was also observed at the termination of lineage two, suggesting that alveolar macrophages begin to express *Spp1* after infection. Overall, the gene expression profiles observed in the monocyte/macrophage subsets indicated notable shifts in differentiation states upon infection, with the striking emergence of an Spp1^+^ macrophage population that displays high expression of fibrotic genes.

### *Coccidioides* is associated with neutrophils and Spp1^+^ macrophages *in vivo*

**Fig 5:**
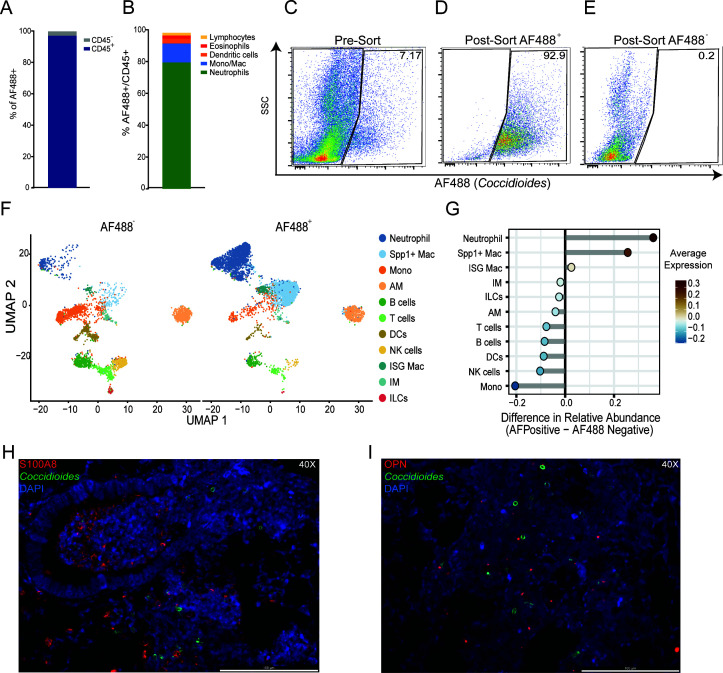
Neutrophils and macrophages associated with *Coccidioides*. Mice were infected intranasally with 1 × 10^6^ AF488-labeled *Coccidioides*, and lungs were harvested 24 hpi and processed for flow cytometric and scRNAseq analysis. Percentage of AF488^+^ cells isolated from the lungs that are CD45^+^ and CD45^–^ (**A**). Percentage of AF488^+^ cells corresponding to the indicated immune cell subtypes (**B**). Flow plots indicate the pre-sort (**C**) and post-sort purities of AF488^+^ (**D**) and AF488^–^ (**E**) populations. Uniform manifold approximation and projection (UMAP) visualization of cell clusters identified in each experimental group (**F**). Relative cellular proportions in AF488^+^ vs AF488^–^ populations (**G**). Immunohistochemistry (IHC) of lung sections co-visualizing AF488-labeled *Coccidioides* with neutrophils (S100A8^+^) (**H**) or cells expressing Osteopontin (OPN) (**I**). Images were taken at 40× magnification and counterstained with DAPI. *n* = 3 uninfected and *n* = 8 infected for flow cytometry. *n* = 3 pooled per condition for sequencing. IHC *n* = 3 per condition. Representative images are shown from a single animal.

Immune responses against fungal infections involve direct interaction with fungi, activation of immune cells, and the release of paracrine signals to alert and recruit other immune cells to the site of infection. To probe the fungal-contact dependency of the cellular responses in the lung, we employed fluorescently labeled fungal spores to separately assess responses of cells directly in contact with *Coccidioides* vs bystander cells. We adapted a fluorescent labeling scheme previously used to label *Bacillus anthracis* spores with an Alexa Fluor 488 (AF488) amine-reactive dye (25). AF488 robustly and uniformly labeled arthroconidia without impacting fungal viability (Fig. S5A and B). AF488-labeled *Coccidioides* was detected interacting with macrophages *in vitro* via fluorescence imaging and flow cytometry (Fig. S5C and D). Flow cytometric analysis of lungs from mice infected with AF488-labeled *Coccidioides* revealed that the majority (>95%) of the cells interacting with *Coccidioides in vivo* were CD45^+^ immune cells (Fig. 5A; Fig. S5E). Approximately 80% of the total CD45^+^ AF488^+^ population were neutrophils (Fig. 5B; Fig. S5E and F). The second most abundant cell type was monocytes/macrophages (AM, IM, and monocytes), collectively representing approximately 12% of the CD45^+^ AF488^+^ population. The remaining 8% were composed of eosinophils, dendritic cells (DCs), and lymphocytes. The flow cytometry data were in line with scRNAseq results (Fig. 1C), which demonstrated that neutrophils and Mono/Mac cells were highly enriched in infected lungs and, furthermore, were the most likely cell types to interact with fungal spores *in vivo* 24 hpi.

Next, we employed scRNAseq to elucidate gene expression changes upon contact with *Coccidioides*. Cells were harvested from lungs 24 hpi as before and enriched for immune cells by bead sorting for CD45^+^ cells. Cells were then sorted into AF488^–^ and AF488^+^ populations using fluorescence-activated cell sorting (FACS), resulting in post-sort purities of >99% and 92.9% for AF488^–^ and AF488^+^ populations (Fig. 5C through E). scRNAseq was then performed on AF488^–^ and AF488^+^ samples, and unsupervised clustering of the data resulted in the identification of 11 cell clusters (Fig. 5F; Fig. S5G). Consistent with flow cytometry data (Fig. 5B; Fig. S15E and F), the relative abundance comparison between sequenced AF488^+^ and AF488^–^ populations indicated that neutrophils and Spp1^+^ macrophages were the most enriched cell types in the AF488^+^ sample (Fig. 5G). The relative abundance of other immune cell types, such as B, T, NK, and dendritic cells, also aligned with flow cytometry data, confirming that these cell types are not frequently associated with *Coccidioides in vivo* 24 hpi (Fig. 5B and G). Interestingly, monocytes were the cell population most highly enriched in the AF488^–^ sample. The corresponding high frequency of monocytes in the AF488^–^ sample and high frequency of Spp1^+^ macrophages in the AF488^+^ sample suggested that monocytes may be differentiating into Spp1^+^ macrophages upon contact with *Coccidioides*. This is further supported by the trajectory analysis in Fig. 4D, which indicates that monocytes move toward the Spp1^+^ lineage upon infection. Immunohistochemistry further confirmed the interaction of *Coccidioides* with neutrophils and Osteopontin (OPN)^+^ cells *in vivo* (Fig. 5H and I). AF488^+^ *Coccidioides* were visualized surrounded by neutrophils (S100A8^+^; Fig. 5H). OPN, the protein produced by the *Spp1* gene, staining is observed intracellularly (purple) and extracellularly (red). OPN^+^ cells are seen surrounding *Coccidioides*, and OPN^+^ localization on the cell surface is oriented toward *Coccidioides* (Fig. 5I). Collectively, these data demonstrate that neutrophils and monocyte/macrophage cells associate with *Coccidioides* rapidly upon infection and suggest a novel mechanism by which monocytes differentiate into Spp1^+^ cells upon contact with *Coccidioides*.

### *Spp1* expression is contact dependent

**Fig 6:**
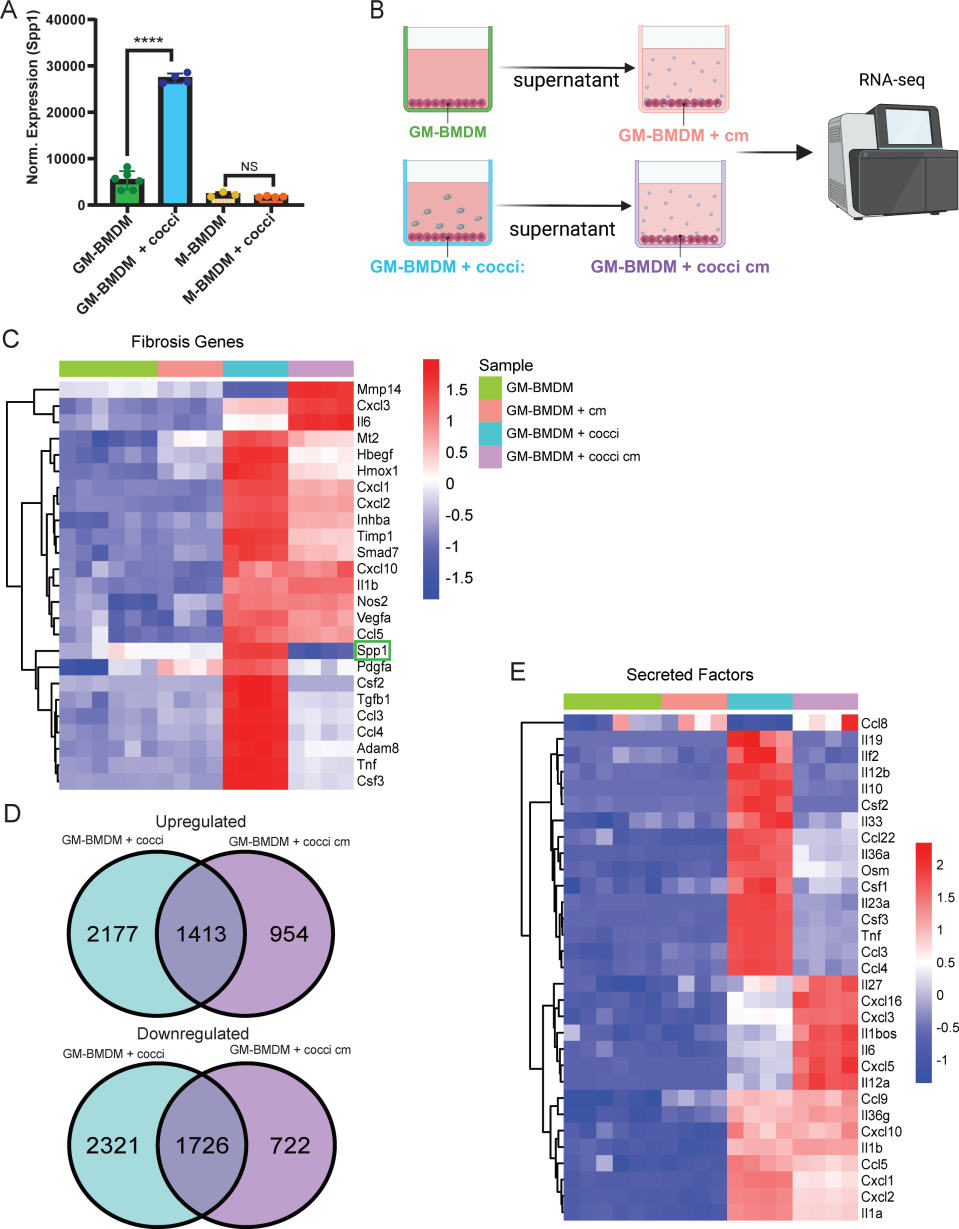
*Spp1* expression is dependent on contact with *Coccidioides*. BMDM were differentiated with GM-CSF (GM-BMDM) or M-CSF (M-BMDM) and exposed to 500,000 *Coccidioides* arthroconidia. RNA was isolated 24 hpi, and RNA sequencing was performed. Normalized expression of *Spp1* (**A**). Schematic of conditioned media experiment (**B**). Heat map depicting expression of selected fibrosis-related genes (**C**). DEGs between stimulated and unstimulated conditions (**D**). Heat map depicting expression of selected cytokine/secreted factor genes (**E**). ****P < 0.0001.

To verify that Spp1^+^ macrophage differentiation is contact dependent, macrophages were exposed to *Coccidioides in vitro* directly or indirectly via conditioned media (cm), and expression of the gene signature was evaluated by RNA-seq. Macrophage colony-stimulating factor (M-CSF) and granulocyte-macrophage colony-stimulating factor (GM-CSF) are both commonly used to induce macrophage differentiation from bone marrow *in vitro*, resulting in macrophages with distinct phenotypic characteristics (26, 27). We differentiated macrophages with each cytokine, then exposed them to *Coccidioides in vitro* to determine which cells would respond to fungal infection by upregulation of *Spp1*. Overnight incubation of GM-CSF differentiated bone marrow-derived macrophages (GM-BMDM) with *Coccidioides* arthroconidia resulted in robust induction of *Spp1* expression (Fig. 6A), whereas exposure of M-CSF differentiated bone marrow-derived macrophages (M-BMDM) to *Coccidioides* resulted in significant cell death, as well as no induction of *Spp1* in surviving cells (Fig. 6A).

Next, to determine if *Spp1* induction was contact dependent, fresh GM-BMDM were exposed to cm collected from control GM-BMDM or GM-BMDM incubated with *Coccidioides* (GM-BMDM + cocci), and RNA-seq was conducted on RNA isolated from GM-BMDM, GM-BMDM + cm, GM-BMDM + cocci, and GM-BMDM + cocci cm (Fig. 6B). Fibrosis-related genes identified as highly expressed in Spp1^+^ macrophages *in vivo* (Fig. 4C) were evaluated within each condition. Limited expression was observed in both control conditions, as expected (Fig. 6C). Robust upregulation of fibrosis-related genes was observed in macrophages in contact with *Coccidioides*, including *Spp1*, *Tgfb1*, *Timp1*, *Adam8*, and *Inhba* (Fig. 6C). While some overlap in gene expression patterns between cells in direct contact with *Coccidioides* and those exposed indirectly via cm was observed, *Spp1* expression was absent in the GM-BMDM + cocci cm condition, and expression of most fibrotic genes was higher in the GM-BMDM + cocci vs GM-BMDM + cocci cm condition (Fig. 6C), indicating that *Spp1* expression, as well as the expression of many fibrotic genes, was dependent upon contact with *Coccidioides*.

GM-BMDM + cocci cm initiated robust changes in gene expression, with 2,367 and 2,448 genes significantly up- or downregulated, respectively (Fig. 6D). Thus, we can infer that direct exposure of GM-BMDM to *Coccidioides* resulted in the release of secreted factors that activated GM-BMDM *in trans*. To probe the landscape of the secretome of GM-BMDM + cocci, we analyzed expression of a panel of secreted factor genes (Fig. 6E). Contact with *Coccidioides* resulted in the upregulation of many pro-inflammatory cytokine genes, including *Il33*, *Il12b*, *Csf1*, *Csf2*, *Il23a*, *Tnf*, *Ccl3*, and *Ccl4*. Interestingly, while some overlap in cytokine gene expression was observed between conditions, including *Il1b*, *Cxcl10*, *Ccl5*, *Cxcl1*, and Cxcl2, distinct patterns of cytokine gene expression were observed in GM-BMDM + cocci vs GM-BMDM + cocci cm conditions. Notably, robust *Il6* expression was observed only within the GM-BMDM + cocci cm condition. Taken together, these data confirm that direct contact with *Coccidioides* is necessary for the induction of *Spp1* expression in macrophages, and furthermore, this contact promotes robust expression of fibrosis-related genes as well as a panel of cytokines that in turn robustly stimulate a distinct activated phenotype *in trans*.

## DISCUSSION

The lung microenvironment presents a complex and dynamic space where the immune response to *Coccidioides* infection unfolds. This response is orchestrated by a range of innate immune cells and is influenced by the unique interactions between the pathogen and host that have not been fully characterized at the cellular and molecular level. Using a comprehensive transcriptomic approach, we have dissected the immediate cellular dynamics triggered by a high dose of *Coccidioides* and identified pivotal cellular players initiating the antifungal immune response. The immune response to high-dose attenuated *C. posadasii* Δcts2/Δard1/Δcts3 24 hpi is characterized by the rapid recruitment and activation of neutrophils and OPN^+^ cells that aggregate around *Coccidioides* and differentiate into PD-L1^+^ neutrophils and Spp1^+^ macrophages. Beyond classic inflammatory cytokines, pathways linked to fibrosis and immune suppression are also engaged within this early timeframe. Chemotaxis and proliferation gene signatures suggest that innate immune populations are recruited to the lung and actively differentiate in response to localized signaling. This early immune activation is crucial for controlling the initial fungal invasion and setting the stage for subsequent adaptive immune responses, but it may also drive inappropriate inflammation, damage, and fibrosis.

To date, there have been very few studies that have probed the interactions of resident non-immune cells of the lung and *Coccidioides*. In our study, modest transcriptional changes were detected among the non-immune cells within the lung during infection, although these cells are critical for maintaining homeostasis and barrier function during infection with other fungal pathogens (21, 28–32). Within this lung subset, an increase in AT2 epithelial cells relative to other non-immune cell types was observed. The main functions of AT2 cells are the production and secretion of surfactant and as progenitors that replenish epithelial cells after lung injury (33). Expansion of this population suggests an attempt to prevent fungal lung entry using surfactant or damage to the epithelium by *Coccidioides* entry and growth. Recent evidence suggests that AT2 cells are an important source of GM-CSF in the lung (34), which is intriguing given the dependence on GM-CSF of Spp1^+^ macrophage differentiation in response to *Coccidioides* exposure in our *in vitro* studies, and suggests that AT2 expansion may be supporting Spp1^+^ macrophage differentiation. Fibroblasts exhibited enrichment in fibrosis-related pathways, which may suggest crosstalk with pro-fibrotic macrophages that emerge after infection. Our data demonstrated very few non-immune cells directly interacting with the fungus at 24 hpi; therefore, the changes observed in host transcription may largely be a response to secreted factors at this time point. Future studies should clarify the role of fungal-host interactions of non-immune cells at even earlier time points to gain further insight into how these earliest interactions shape downstream responses. The molecular interactions suggest a combined repair and protective response by the barrier cells within the lung following fungal infection, but they also suggest poor barrier activation and a transcriptional profile shifted toward a pro-fibrotic response.

Neutrophils were rapidly recruited to the lung and constituted the largest cellular increase after infection. Surprisingly, the absolute requirement for neutrophils in acute *Coccidioides* control is uncertain. Neutrophils have been shown to robustly respond to arthroconidia and spherules (14, 35) and are required for vaccine efficacy against *Coccidioides* (18), although their depletion does not impact fungal burden in unvaccinated mice (18). During infection, neutrophils produce IL-10, an immunosuppressive cytokine that may reduce iNOS expression, affecting infection responses (15, 17, 36). Mice lacking Cxcr2 show fewer neutrophils, lower IL-10 levels, and reduced fungal burden, suggesting that neutrophils play a role in worsening outcomes (37). Here, neutrophils were subclustered into five subsets, with largely overlapping gene signatures, likely indicating active differentiation within the lung microenvironment 24 hpi. The presence of circulating and early neutrophils undergoing differentiation suggests active granulopoiesis in the bone marrow, seeding the lung tissue. Trajectory analysis indicates that circulating and early neutrophils differentiate into PD-L1^+^ neutrophils, a population known to negatively regulate neutrophil activity (38–40). Future studies are needed to clarify how PD-L1^+^ neutrophils shape outcomes during *Coccidioides* infection and to determine if PD-L1^+^ neutrophils arise through fungal contact-dependent mechanisms, similar to Spp1^+^ macrophage differentiation. Interestingly, this population also dominates the lung during wild-type *Coccidioides* infection, although at later stages post-infection (41). Differences in the differentiation kinetics of PD-L1^+^ neutrophils between these studies may be due to fungal dosing or fungal evasion mechanisms that require spherule maturation. Understanding how attenuated *Coccidioides* strains drive PD-L1 expression and associated gene signatures may inform future *Coccidioides* vaccine design, suggesting the need to incorporate more effective adjuvant choices to promote durable Th1 immunity without premature checkpoint engagement.

The second most prominent cellular response observed in our study is the monocyte-derived macrophages that rapidly differentiate within the tissue by upregulating Spp1. Spp1 expression is an emerging biomarker in various diseases, with the presence of Spp1^+^ macrophages being linked to poor prognosis in severe COVID-19, active TB infection, lung fibrotic diseases, and cancer (42–51). Trajectory analysis indicated classical monocytes transition into Spp1^+^ macrophages following *Coccidioides* infection, and *in vitro* experiments indicated that this differentiation is dependent on fungal contact. OPN, produced by the Spp1 gene, is a versatile secreted molecule, inducing pro- and anti-inflammatory responses through several pathways, including the Dectin-1 receptor, which is crucial for recognizing *Coccidioides* (52–54). Future studies are necessary to define the surface molecules and downstream signaling pathways expressed by monocytes that engage with *Coccidioides* and result in Spp1^+^ macrophage differentiation. In a mouse model of *Candida* infection, *Spp1* deficiency completely ameliorated disease lethality (55). In severe COVID-19, elevated OPN plasma levels activated CD14 monocytes and PD-L1^+^ neutrophils, contributing to disease progression (52). Together, these findings suggest that upregulation of *Spp1* may initially represent an attempt to control disease following direct contact with *Coccidioides* but may ultimately be detrimental to infection outcomes. Thus, Spp1^+^ macrophage polarization in *Coccidioides* may represent the beginning of a maladaptive immune response that promotes PD-L1^+^ neutrophil differentiation (56, 57). Additional studies are needed to clarify the role of Spp1^+^ macrophages in disease outcomes; however, Spp1^+^ macrophages also develop in response to virulent *Coccidioides* infection, expanding from 5 to 14 days post infection (dpi) and demonstrating a similar fibrotic phenotype (41). The rapid induction of Spp1^+^ macrophages suggests that anti-fibrotic strategies might be considered early in infection to prevent inappropriate induction of tissue remodeling.

Our analysis of the early immune response to *Coccidioides* infection demonstrated rapid influx and activation of neutrophils, particularly PD-L1-expressing subsets, and the differentiation of monocytes into Spp1^+^ macrophages. An important limitation to consider in the interpretation of our data is that the large inoculum dose of an attenuated strain may induce responses that are not representative of natural infection. Indeed, the large cellular influx at 24 hpi is undoubtedly influenced by the large inoculum dose, whereas cellular influx in wild-type infection is typically seen after spherule rupture beginning at 5 dpi (8). Nevertheless, we find it striking that similar populations of neutrophils and macrophages emerge in our study utilizing a wild-type infection model (41), albeit with a different timeline. Consideration of our two studies together suggests that differentiation of neutrophil and macrophage subtypes is not dependent on the type of fungal body encountered, as the predominant form of the fungus at 24 hpi are arthroconidia or early spherules, whereas at later time points in our study utilizing a low dose of wild type *Coccidioides*, the predominant fungal form is endospores. Future studies should focus on the functional roles of these immune subsets in chronic infection stages and their impact on disease progression. Investigations could include examining the molecular mechanisms driving PD-L1^+^ neutrophil differentiation and activation, determining the specific contributions of Spp1^+^ macrophages to tissue fibrosis, and exploring potential therapeutic interventions to modulate these immune responses. Future studies should also address additional limitations of this study, such as the exclusive use of female mice of a highly susceptible strain. Expanding to include male mice of strains displaying variable susceptibility will inform how early immune responses shape infection outcomes and may highlight differences between productive vs maladaptive responses. Additionally, studies utilizing virulent fungal strains and varying dosages could provide further insights into the kinetics and severity of immune responses, ultimately informing better strategies for prevention and treatment.

## MATERIALS AND METHODS

### Fungal culture

*C. posadasii* (Δ*cts2*/Δ*ard1*/Δ*cts3*) derived from patient isolate C735 was used for all infections (NR-166 BEI Resources, Manassas, VA, USA). Liquid 2× Glucose 1× Yeast Extract (2× GYE) media (Fisher Scientific, Hampton, NH, USA) with 50 mg/mL hygromycin B (Invitrogen CAT# 10687010) was used to grow *Coccidioides* mycelia in a shaking incubator at 37°C for a week. Mycelia was then streaked onto solid 2× GYE agar and desiccated in an incubator at 30°C for a month to disassociate mycelia into individual arthroconidia. To harvest arthroconidia, the white fuzzy growth (arthroconidia) was scraped off with 1× phosphate buffered saline (PBS; VWR) and filtered through a 100 µM mesh filter. *Coccidioides* was then vortexed for 1 min to dissociate the arthroconidia and centrifuged at 12,000 × *g* for 8 min with a break at room temperature. The pellet was washed and vortexed with 30 mL of PBS and centrifuged again. After the second spin, 10 mL 1× PBS was added to the pellet, and the working stock was stored at 4°C. Fresh arthroconidia stocks were prepared monthly. See Mead et al. for detailed protocol (58).

### Arthroconidia fluorescent labeling

Alexa fluor 488 (AF488, ThermoFisher, Cat #: A20000) was used to label arthroconidia spores as previously described (25). Briefly, AF488 was resuspended in 100 µL dimethyl sulfoxide for the final concentration of 10 mg/mL, aliquoted, and stored at −20°C. To label *Coccidioides*, 1 mL of fresh 1 M NaHCO_3_ and 5 µL AF488 were combined in a 15 mL tube along with 6 × 10^6^ arthroconidia and 1× PBS added to a final volume of 10 mL. The mixture was incubated at room temperature for 3 h, then centrifuged at 12,000 × *g* for 8 min with break off at room temperature. The pellet was then washed with 15 mL PBS and centrifuged again, then resuspended to a final concentration of 1–10 × 10^6^ cells/40 µL.

### Mouse infections

Female C57BL/6 mice (6–8 weeks old, JAX #000664, The Jackson Laboratories, Bar Harbor, ME, USA) were used for all animal experiments. Animals were housed in an Association for Assessment and Accreditation of Laboratory Animal Care-accredited facility. Mice were infected intranasally with 1 × 10^6^ arthroconidia (with AF488 label or unlabeled) in 40 µL (20 µL per nostril) 1× PBS while under anesthesia (4%–5% isoflurane (Covetrus) in 100% oxygen). For tissue harvest, animals were anesthetized under isoflurane, and the whole animal was perfused with 20 mL sterile PBS containing 50,000 U/L sodium heparin (Sigma) via the left ventricle.

### Lung isolation and preparation of single cell suspensions

Following euthanasia and perfusion, lungs were removed and placed in a 100 mm petri dish containing 5 mL of digestion buffer (DMEM/F12 [Thermo Fisher] + 100 µg/mL DNase I [Roche cat# 11284932001] and 3 mg/mL collagenase 1 [Worthington cat# LS004197]) and minced with scissors into pieces approximately 1 mm in diameter. Minced pieces were transferred to a 15 mL conical tube, and the petri dish was rinsed with another 5 mL digestion buffer, which was then added to the 15 mL tube. Lung homogenate was incubated for 30 min in a 37°C shaking incubator at 150 rpm, with cells triturated with a 10 mL pipette at 15 min. At 30 min, the supernatant was removed and transferred to a 50 mL conical tube and placed on ice. A total of 10 mL of fresh digestion buffer was then added to the remaining lung homogenate and incubated for another 30 min with shaking as above. Following the second 30 min incubation, the remaining unhomogenized tissue was pipetted into a 70 µm mesh filter and manually dissociated with the flat end of a pellet pestle. Equal volume DMEM/F12 + 10% fetal bovine serum (FBS, Thermo Fisher) was added to the cell suspension to neutralize digestion enzymes and pelleted by centrifugation at 500 × *g* at 4°C for 8 min. Red blood cells were removed by incubation in ammonium-chloride-potassium lysis buffer (Thermo Fisher) for 5 min at room temperature. Cells were then resuspended in DMEM/F12 + 10% FBS and counted on a Countess II (Thermo Fisher) automated cell counter prior to downstream analysis.

### Preparation of cells for scRNAseq

For whole lung sequencing, 3 × 10^6^ cells were fixed per mouse using 10× Genomics Fixation Buffer according to the manufacturer’s instructions, and fixed cells were stored at −80°C prior to sequencing. When thawed, fixed cells were counted again using a Countess 3 (Thermo Fisher), and 500,000 cells each from three mice were pooled per reaction. For sequencing of sorted cells, following generation of single cell suspensions, immune cells were enriched using LS columns (Miltenyi Biotec) for magnetic-activated cell sorting (MACS) separation using anti-CD45 magnetic microbeads (Miltenyi Biotec, 130-052-301) according to the manufacturer’s instructions, followed by resuspension in 10× Genomics Fixation Buffer and fixed overnight at 4°C. Fixed cells were pooled and sorted using a BD FACSAria Fusion and BD FACSMelody Cell Sorter to collect AF488^–^ or AF488^+^ populations. BD FACSDiva Software Version 8.0.1 and BD FACSChorus Software Version 2.0 (Application Data Version 1.1.20.0) were used during acquisition to define the sorted populations. Sorted cell suspensions were resuspended in 10× Genomics Quenching Buffer and glycerol according to the manufacturer’s instructions and stored at −80°C prior to sequencing.

### scRNAseq library preparation

Single-cell RNA sequencing was performed using the 10× Genomics Chromium Single Cell Fixed RNA Profiling (Flex) Kit (cat# 100475), following the manufacturer’s protocol. Following the generation of single-cell suspensions as described above, 3 × 10^6^ cells per lung sample were fixed using 10× Genomics fixation buffers supplemented with paraformaldehyde to a final working solution of 4% according to the manufacturer’s instructions and then stored at −80°C 10× Genomics long-term storage solution prior to library preparation. Each reaction was inclusive of three mice validated for fungal infection by plaque assay and pooled at 500,000 cells per mouse. Probe hybridization was then performed overnight, followed by pooling before loading onto the Chromium X Controller (10× Genomics) to generate single-cell gel bead-in-emulsions. Reverse transcription, cDNA amplification, and library amplification were performed according to the manufacturer’s recommendations. Quality control measures, including quantification and size distribution analysis, were performed using an Agilent Tape Station. Sequencing libraries were then sequenced on an Illumina NextSeq 2000 platform, producing paired-end reads. Data processing, including alignment, barcode assignment, and gene expression quantification, was performed using the Cell Ranger software pipeline (v 7.2.0) from 10× Genomics (59). Alignments were carried out using the reference genome package refdata-gex-mm10-2020-A for GRCm38 (mm10), provided by 10× Genomics. Additionally, the “Chromium_Mouse_Transcriptome_Probe_Set_v1.0.1_mm10_2020-A” probe set from 10× Genomics was utilized.

### Flow cytometry

Following the generation of single-cell suspensions as described above, 1 million cells per condition were incubated with the following antibodies: CD11c PE Cy7 (BD, CAT# 558079, clone HL3, 1:500), SiglecF PE (Invitrogen, CAT# 12-1702-82, clone 1RNM44N, 1:500), CD11b BV 510 (Biolegend, CAT# 101263, clone M1/70, 1:500), Ly6C APC (#BD, CAT# 560595, clone AL-21, 1:500), and Ly6G BV711 (BD, CAT# 563979, clone 1A8, 1:500). Samples were incubated with antibody cocktail for 30 min at 4°C, washed, fixed with 4% paraformaldehyde for 1 h, then washed, and resuspended in 200 µL of PBS for flow cytometry. Samples were acquired using the BD FACSAria Fusion Flow Cytometer utilizing the BD FACSDiva Software Version 8.0.1. Flow cytometry results were analyzed using FlowJo v10.10 Software (BD Life Sciences). All immune cell types were first gated on CD45 and further identified by expression of the following markers: lymphocytes: CD11b^–^CD11c^–^, AM: CD11b^lo^SiglecF^+^, eosinophils: CD11b^hi^SiglecF^+^, neutrophils: CD11b^+^Siglec F^–^Ly6G^+^, DCs: CD11c^+^MHC II^+^, monocytes: CD11b^+^SiglecF^–^Ly6G^–^ MHC II^–^Ly6C^+^, IM: CD11b^+^SiglecF^–^Ly6G^–^ MHC II^–^Ly6C^–^. Sequential gating strategy is visualized in Fig. S5E.

### Immunohistochemistry

Lungs for immunohistochemical staining were collected from infected mice at 24 hpi. After euthanasia and perfusion, the lungs were inflated and immersed in 10% neutral buffered formalin at 4°C for 3 days with mild agitation. Lung samples were then paraffin embedded and sectioned into 5 µM thick sections using a Leica RM2255 Microtome. The sections were deparaffinized using xylene and hydrated in a series of alcohol solutions. Antigen retrieval was performed at 65°C with Tris-EDTA (Abcam, ab93684), and non-specific sites were blocked with CAS-block (008120, Thermo Fisher Scientific). The sections were incubated with primary antibodies S100A8 (Abcam, ab92331) and OPN (AF808, R&D), followed by secondary antibodies (A11037 and A11058, Thermo Fisher Scientific). Finally, the slides were mounted with ProLong Gold Antifade Mounting with 4',6-diamidino-2-phenylindole (DAPI, ThermoFisher, CAT# P36935) and imaged using a Leica DM4 B microscope with 20× and 40× objectives.

### scRNAseq data analysis

#### Full data set analysis

Analysis was initially performed using Seurat (v4.3.0) (60) and R (v4.3.2) but transitioned to Seurat (v5.1.0) (61) and R (v4.4.0). Seurat objects were created for each sample using the CreateSeuratObject function with default parameters and subsequently merged into a single object. For the analysis, we utilized uninfected lung sample data from the D0 timepoint, as described in Davalos et al. (41). Quality control of the data sets involved retaining cells with at least 500 counts, cells with at least 300 expressed genes, and cells with less than 10% mitochondrial genes (nCount_RNA ≥ 500, nFeature_RNA ≥ 300, and percent_mt < 10). We excluded lowly expressed genes by retaining only those genes that were expressed in a minimum of 10 cells.

#### Broad analysis of the entire data set

Data were normalized using the “NormalizeData” function using default parameters. Highly variable genes (HVGs) were identified using the “FindVariableGenes” function. Before dimensional reduction, the data were scaled with the effects of cell counts and mitochondrial percentages regressed out using “ScaleData” (vars.to.regress = c [“percent_mt,” “nCount_RNA”]). After performing principal component analysis (PCA) using the “RunPCA” function, the top 50 principal components were selected for downstream integration, clustering, and dimension reduction.

Data integration was performed using Harmony (v1.2.0) (62) with the grouping variable set to “orig.ident,” which contained both samples. Clustering analysis was performed using FindNeighbors and FindClusters, with the reduction parameter set to “harmony” and resolution of 0.4. Subsequently, a non-linear dimensionality reduction was conducted using uniform manifold approximation and projection (UMAP), with “RunUMAP” (reduction = “harmony”; umap.method = “uwot”).

To distinguish different cell populations, cell annotation was performed using differential expression, known marker genes, and a scRNAseq resource LungMAP (63) was used. Labels were transferred (lineage_level1, lineage_level2, and celltype_level2) from the mouse LungMAP scRNAseq object (64) by using two functions “FindTransferAnchors” (reference = lungmap; query = ourdata) and “TransferData” (dims = 1:30). Next, “FindAllMarkers” (only.pos = true) was used to identify cluster marker genes and annotate cell clusters using known marker genes. The predicted labels and the label scores from LungMAP were overlaid to confirm appropriate cell labeling across all populations.

Lastly, the relative abundance of cell types was calculated by determining the proportion of each cell type within each sample. The difference in proportions between two samples was then computed for each cell type. These differences were used to visualize changes in cell type abundance between the samples.

### Neutrophil subset

To analyze neutrophils and their various subsets, we followed the same approach used for the entire data set with a few modifications. Given the smaller size of the neutrophil subset, we identified 2,000 HVGs. For dimension reduction, integration, and clustering, 10 principal components and a cluster resolution of 0.5 were used. For marker gene identification, the same method was employed as described above in 2.10b. To identify biologically relevant pathways, the top 100 DEGs that met our filtering criteria (*P*-value adjusted < 0.05 and average log2 fold change ≥ 1) were selected. To perform enrichment analysis, the clusterProfiler (v4.12.0) (65) “compareCluster” function with biological process ontology (ont = BP) was utilized. To perform a trajectory analysis, we used Slingshot (v2.12.0) (24) “slingshot” (extend = *n*, stretch = 0, start.clus = Circulating Neu.) function on the UMAP embedding. Lastly, we utilized functions from the Single-Cell Pipeline (v0.5.6) package to fit generalized additive models (GAMs) for analyzing gene expression along pseudotime in scRNAseq data (66). Briefly, a GAM was applied to each gene, with gene expression as the dependent variable and pseudotime as the independent variable, incorporating smooth functions to capture non-linear relationships. This approach enabled the detection of changes in gene expression over pseudotime. We generated a heatmap of the dynamic features along the pseudotime trajectory using functions “RunDynamicFeatures” (n_candidates = 2000, seed = 2024, and minfreq = 0), and “DynamicHeatmap” (use_fitted = TRUE, n_split = 3, r.sq = 0.09, dev.expl = 0.1, and num_intersections = NULL).

### Myeloid subset

To further analyze myeloid cells and their various subsets, we followed the same approach used for the entire data set (2.10b) with a few modifications. Given the smaller size of the myeloid subset, we identified 2,000 HVGs. For dimension reduction, integration, and clustering, we used 30 principal components and a cluster resolution of 0.8. For marker gene identification, we employed the same method as described above. Calculation of relative abundance was performed as stated above. Trajectory analysis was performed using Monocle2 (v2.32.0) (67) with the following sequence of functions and parameter adjustments: “as.CellDataSet,” “estimateSizeFactors,” “estimateDispersions,” “reduceDimension” (reduction_method = “DDRTree” and pseudo_expr = 1), and “orderCells” (reverse = FALSE).

### Sorted AF488^−^ and AF488^+^ cells

For the analysis of the sorted data, the same approaches were used as detailed in the broad analysis of the entire data set section, with the following modifications. Seurat objects for each sample, using CreateSeuratObject with default settings, were constructed and merged into a unified object. To ensure data quality, we implemented several filters: retaining cells with at least 200 and less than 5,000 counts, at least 200 and less than 5,000 expressed genes, and less than 10% mitochondrial genes (200 ≥ nCount_RNA ≤ 5000, 200 ≥ nFeature_RNA ≤ 5000, and percent_mt < 10). Again, our analysis was restricted to genes that expressed in a minimum of 10 cells. We identified 3,000 HVGs. For dimension reduction, integration, and clustering, 40 principal components and a cluster resolution of 0.4 were used. Annotation and calculation of relative abundance were performed as stated above.

### *In vitro* macrophage differentiation and infection

Cells were isolated from bone marrow as previously described (68). After processing to a single-cell suspension, red blood cells were lysed, and cells were counted as described above. A total of 500,000 cells per well were plated in a 12-well tissue culture treated plate in 2 mL DMEM/F12 + 10% FBS supplemented with 100 ng/mL M-CSF (Thermo Fisher, cat# PMC2044) or 100 ng/mL GM-CSF (R&D systems cat# 415 mL) to generate M-BMDM or GM-BMDM, respectively. Macrophages were differentiated for 7 days *in vitro*, with 1 mL media supplementation after 3 days in culture and a half media change on day 5. For direct contact experiments, 500,000 arthroconidia were added to macrophages in DMEM/F12 + 10% FBS and incubated for 24 h. Supernatants were collected, centrifuged at 2,000 × g for 10 min to remove cellular debris, and filtered through a 0.22 µM filter to generate conditioned media. Conditioned media was also collected from mock-infected cells. For conditioned media experiments, GM-BMDM were incubated for 24 h with conditioned media supplemented with an additional 5% FBS from GM-BMDM infected with *Coccidioides* or mock-infected cells. RNA was isolated from all conditions using RNeasy RNA mini kits (Qiagen, cat #74014) according to the manufacturer’s instructions.

### RNA-Sequencing library preparation and analysis

Sequencing library preparation was performed using Illumina Stranded mRNA Prep, Ligation (Illumina, cat # 20040534) according to the manufacturer’s recommendations. Sequencing was performed on an Illumina NextSeq 2000 using P2 reagents. The raw RNA sequencing data were aligned to the mouse genome (mm10) using STAR (69), and mapped reads were counted using featureCounts (70). Subsequently, data normalization was performed using the Trimmed Mean of M-values method (71). DEGs were identified using edgeR (version 4.2.1) (72) in R (version 4.4.1). Genes were considered significantly expressed when their false discovery rate-adjusted *P*-value was <0.05, and fold change was 1.5. Pathway and biological process enrichment analyses were conducted using the ToppGene Suite (73), based on the DEGs identified. Heatmaps were generated using the “pheatmap” package (version 1.0.12) in R.

### Statistics

Data were evaluated using one- and two-way analysis of variance followed by either Tukey’s or Šídák’s multiple comparisons test. Grubbs’ outlier calculations were performed as needed for flow cytometry data. GraphPad Prism version 10.2.2 for Windows was used, GraphPad Software (Boston, Massachusetts, USA).

## Data Availability

The single-cell RNA sequencing and bulk RNA sequencing data have been deposited in the NCBI Gene Expression Omnibus (GEO) under GEO accession numbers GSE275512 (scRNAseq) and GSE275509 (bulk RNAseq).

## SUPPLEMENTAL MATERIAL

Supplemental figures spectrum.00442-25-s0001.pdf Fig. S1 to S5.

Graphical Abstract spectrum.00442-25-s0002.png Graphical abstract.

ASM does not own the copyrights to Supplemental Material that may be linked to, or accessed through, an article. The authors have granted ASM a non-exclusive, world-wide license to publish the Supplemental Material files. Please contact the corresponding author directly for reuse.
