# Supplementary material for: Early immune response to Coccidioides is characterized by robust neutrophil and fibrotic macrophage recruitment and differentiation
Source: Microbiol Spectr. 2025 Jul 24;13(9):e00442-25. doi: 10.1128/spectrum.00442-25 (PMC12403890; doi:10.1128/spectrum.00442-25)
Supplement: Supplemental figures — Fig. S1 to S5. [file spectrum.00442-25-s0001.pdf]

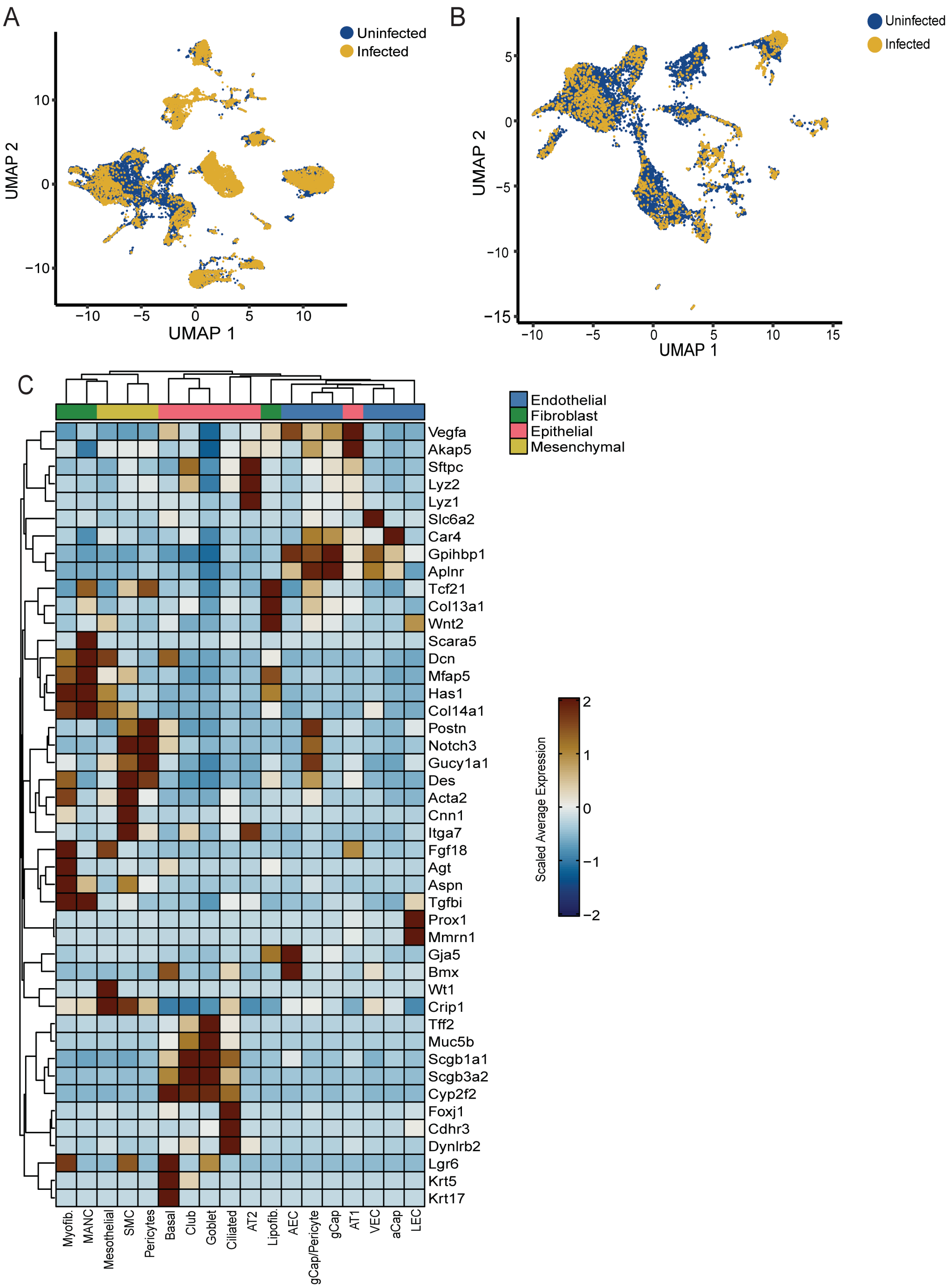

Supplemental Figure 1: UMAPs and heat map of immune and non-immune cells (A) UMAP overlay of all lung cells in the infected and uninfected samples from Figure 1B. (B) UMAP overlay of non-immune lung cells in the infected and uninfected samples from Figure 2A-B. (C) Heatmap of non-immune cells classification and gene signatures from Figure 2.

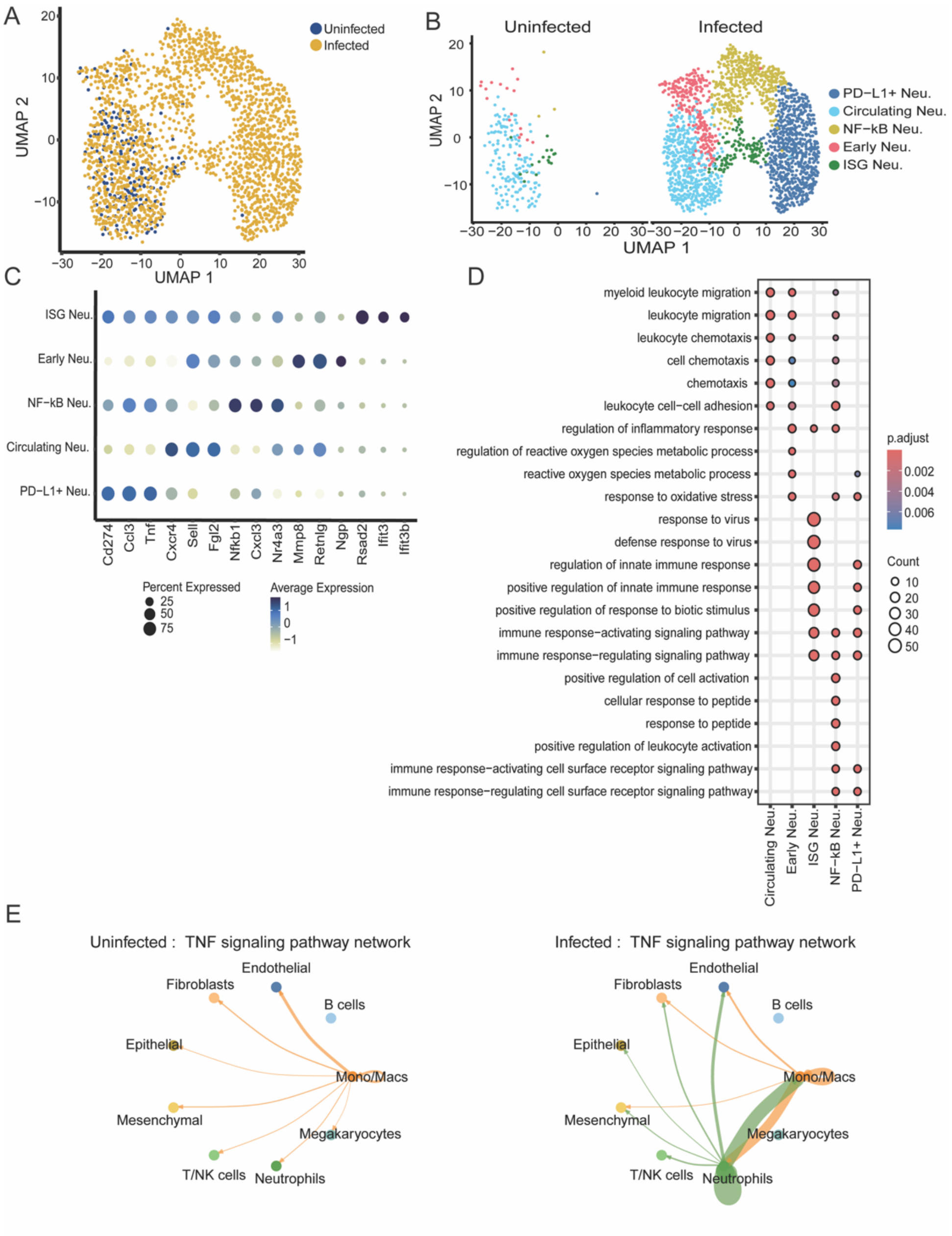

Supplemental Figure 2: Supporting neutrophil data (A) Subtypes of neutrophils overlayed between uninfected and infected. (B) UMAP of uninfected and infected neutrophil subtypes. (C) Dot plot demonstrating top three gene signatures of each neutrophil subclass. (D) GO showcasing increased and decreased pathways associated with each neutrophil subclass. (E) CellChat identified TNF signaling in uninfected and infected samples.

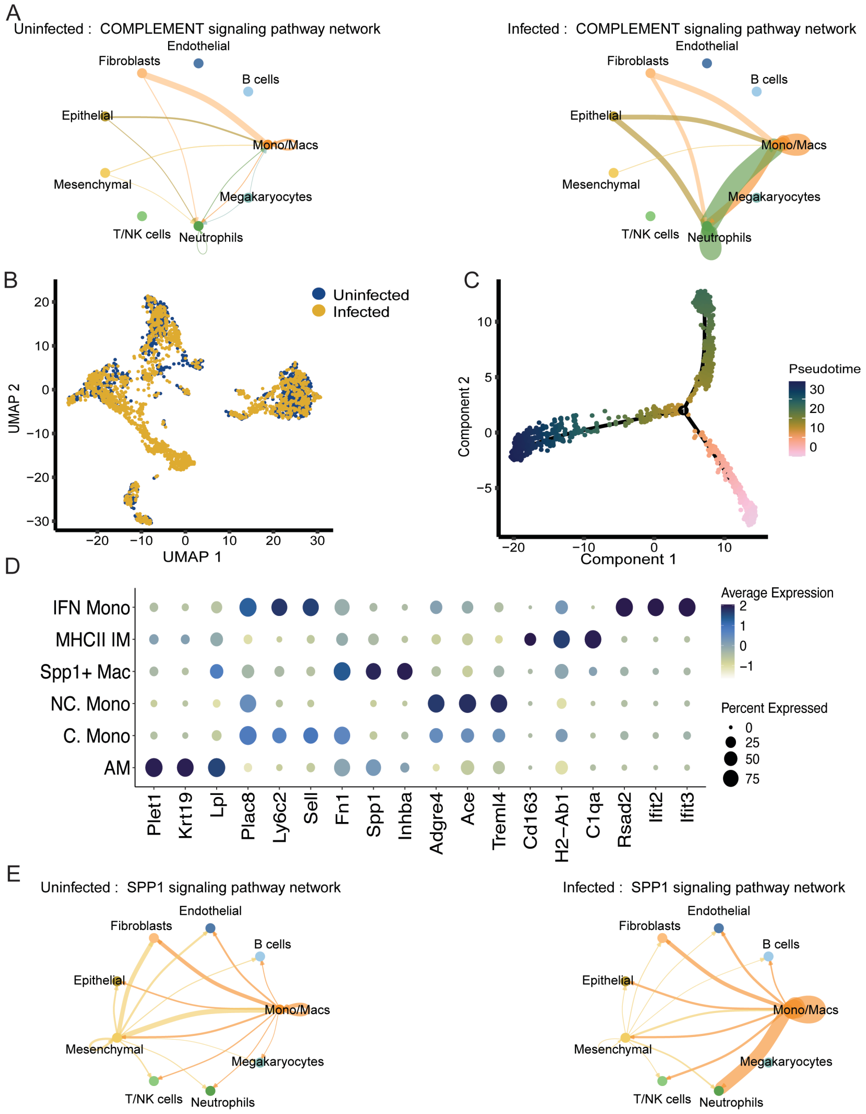

Supplemental Figure 3: Extra macrophage data (A) Complement signaling is through CellChat in uninfected and infected samples. (B) UMAP overlay macrophage subclusters from the uninfected and infected samples. (C) Pseudotime analysis on the macrophage subclusters for trajectory analysis. (D) Dot plot showing the top 3 genes expressed by each macrophage subclass. (E) Spp1 signaling via CellChat in infected and uninfected samples.

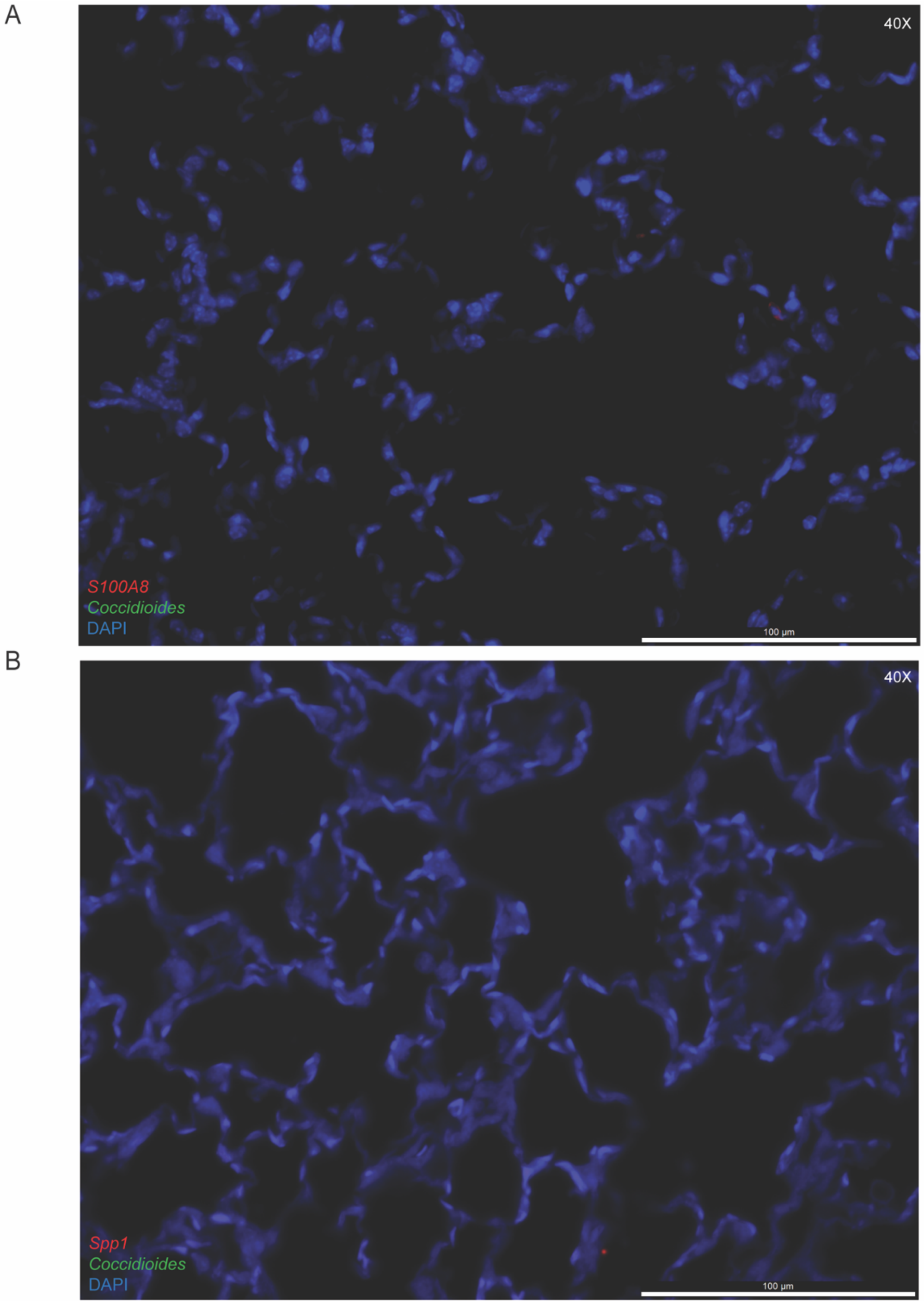

Supplemental Figure 4: Uninfected IHCs (A) Wild type mouse stained with S100A8 and DAPI, image taken at 40X. (B) Wild type mouse stained with Spp1 and DAPI, image taken at 40X.

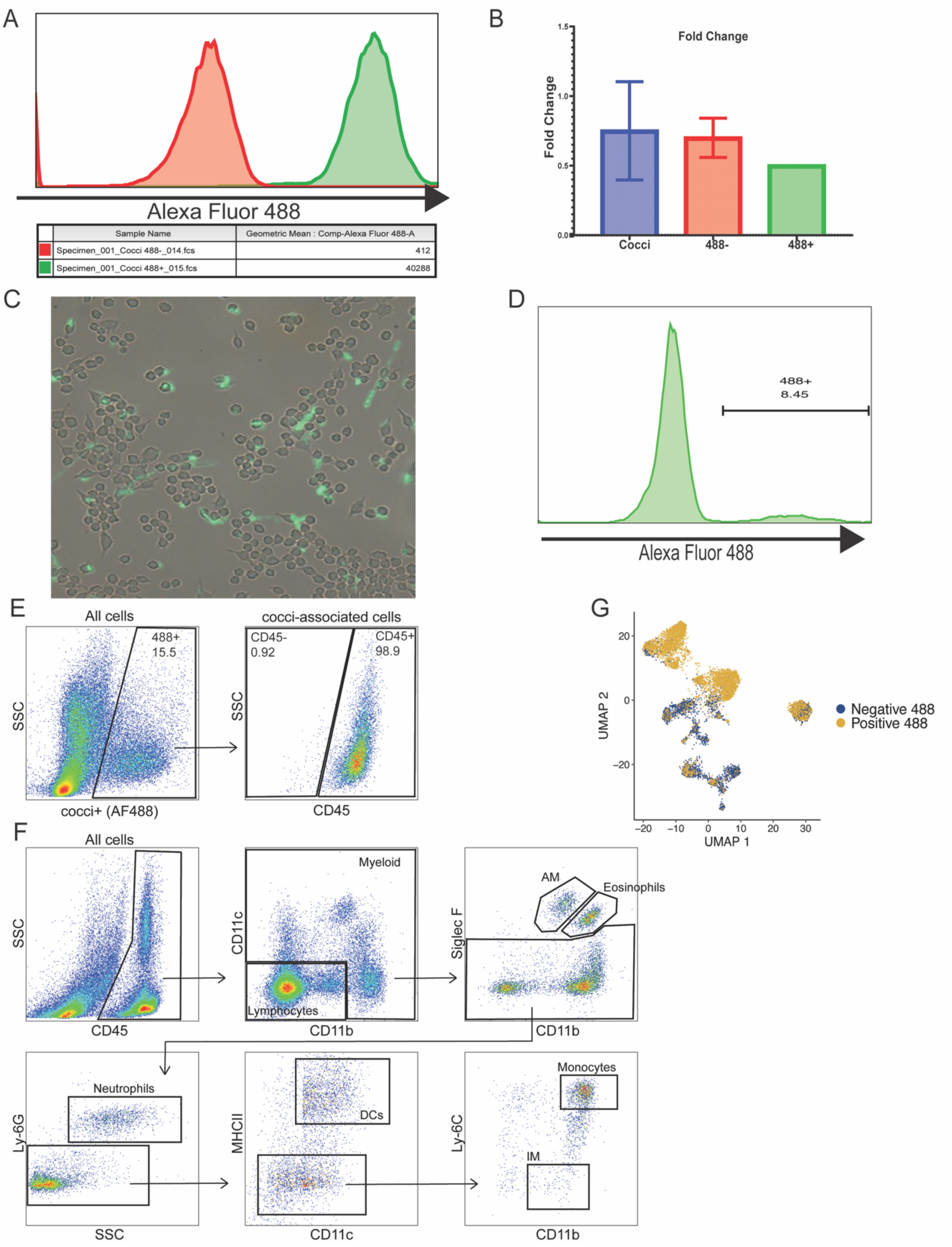

Supplemental Figure 5: AF488 labeling and flow cytometry gating strategy. (A) AF488 labeled Coccidioides is detected by flow cytometry. (B) Viability of Coccidioides is unchanged by the

addition of AF488 label. (C) Labeled Coccidioides co-cultured with RAW 264.7 macrophages are visualized by fluorescence microscopy (C) and detected by flow cytometry (D). (E-F) Gating strategy used to identify $AF488^{+}$ and immune populations.
