# Supplementary figures and images for: Early immune response to Coccidioides is characterized by robust neutrophil and fibrotic macrophage recruitment and differentiation
Source: Microbiol Spectr. 2025 Jul 24;13(9):e00442-25. doi: 10.1128/spectrum.00442-25 (PMC12403890; doi:10.1128/spectrum.00442-25)

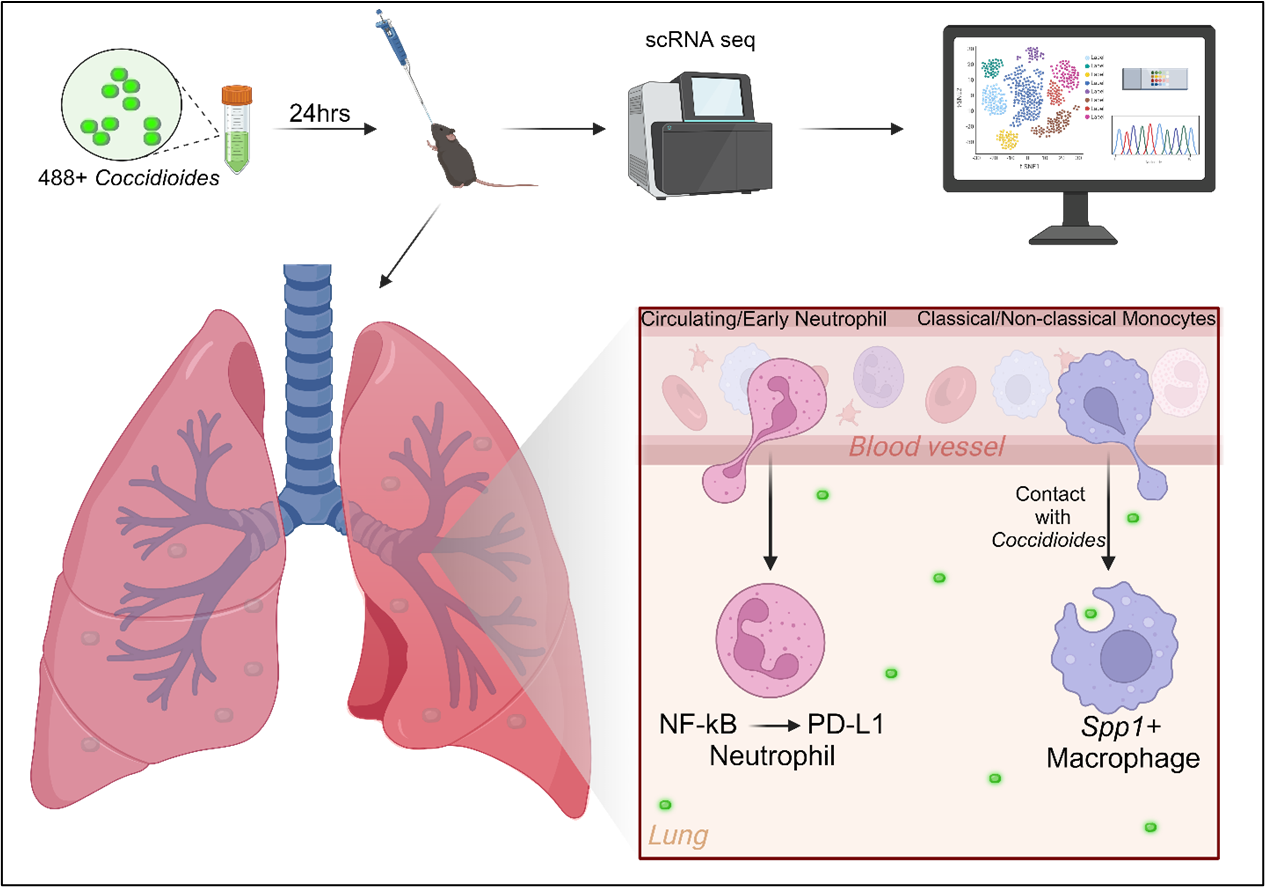

Supplement: Graphical Abstract [file spectrum.00442-25-s0002.png]
